# Knowledge, Attitudes, and Practices of the Population in Jazan Region, Saudi Arabia Regarding Dengue Fever and Its Prevention Measures: A Community-Based Cross-Sectional Study

**DOI:** 10.3390/ijerph192416812

**Published:** 2022-12-14

**Authors:** Anas Elyas Ahmed, Mohammed A. Almarhabi, Mohammed O. Shami, Alhassan Ali Alhazemi, Hassan M. Alsharif, Ali Essa Abu Hayyah, Wessam Ahmed Alhazmi, Mohammed A. Alfaifi, Abdulaziz Q. Abdali, Mohammed S. Alshihri, Ali H. Alhazmi, Halimah A. Qasem, Mazen Ahmed Alhazmi, Siddig Ibrahim Abdelwahab

**Affiliations:** 1 Faculty of Medicine, Jazan University, GGGD6622, Jazan 45142, Saudi Arabia; 2 Tabuk Pharmaceuticals, Jazan 48635, Saudi Arabia; 3 Medical Research Centre, Jazan University, GGGD6622, Jazan 45142, Saudi Arabia

**Keywords:** knowledge, attitude and practice, dengue fever, prevention measures, Saudi Arabia

## Abstract

In previous studies, there was an apparent lack of health education about dengue fever (DF) among the Saudi population. Therefore, we conducted this study to assess the knowledge, attitude, and practices (KAP) about dengue fever among the Jazan region population, which is one of the most prevalent diseases in the region in Saudi Arabia (KSA). This was a cross-sectional and community-based study. The adult population was divided into governorates according to the regions that were close to each other, and then a convenient stratum was selected from each region. Next, random sampling was applied. Data were collected using a self-administered questionnaire. Exclusion criteria were young people (<18 years old) and health workers. Data analysis was performed using descriptive statistics, the Pearson’s correlation coefficient, and multiple linear regression. Of the 392 participants in this cross-sectional study, 59.18% were male, 76.28% were aged 18–35 years, 72.96% had a university degree, and 63% had a monthly income of less than SAR 5000 (USD1 = 3.76). The scores (mean ± SD) for KAP regarding DF among the responders were 22.77 ± 7.9, 22.68 ± 7.24, and 25.62 ± 9.4, respectively. KAP constructs were positively correlated according to the Pearson’s coefficient. In multiple linear regression analysis, males were favorably and substantially linked with attitude score (β = 2.76, *p* = 0.001) and negatively associated with practice score (β = −2.45, *p* = 0.023). No-degree participants scored lower on knowledge (β = −2.78, *p* = 0.003). There is potential for more research in Saudi Arabia to increase the generalizability to reduce the impact of dengue epidemics.

## 1. Introduction

Dengue fever (DF) is a widespread arboviral disease transmitted by mosquitoes [1]. There are 96 million symptomatic cases annually and 3.9 billion people at risk of infection worldwide. The spread of DF in the Kingdom of Saudi Arabia (KSA) from 1993, when the first case was recorded, until today, when the cases reached 13.6 per 100,000, has led the Saudi Ministry of Health (MOH) to consider the area as endemic. Dengue fever is found along the western coast of the KSA including the Jazan region located in the southwest of Saudi Arabia, where the ideal factors are available for the transmission of dengue infection such as a hot, humid, rainy climate and the stagnant water that occurs after rain [2,3]. Additionally, numerous people in villages work as farmers and shepherds. These people may use the farms for sleeping in when there are problems with electricity at home and thus expose themselves to adult female mosquito (vector) bites, especially with animal water basins outside their homes [4,5].

There are four types of dengue virus included in the Flaviviridae family, which are transmitted by the bite of the vector; *Aedes aegypti* and, less likely, *Aedes albopictus* [6]. Most DF patients are asymptomatic, and the symptomatic patient usually complains of non-specific symptoms such as fever, headache, myalgia, arthralgia, nausea, and skin rash [7,8]. DF can develop into a more severe condition, dengue hemorrhagic fever, which may cause death. Morbidity and mortality rates are correlated with abdominal pain, vomiting, a decreased platelet count, and elevated liver enzymes [7,9]. When infection occurs, the person develops lifelong immunity toward the type that caused the infection. There is no specific drug for DF, and the vaccine cannot be given in many places. In 2008, the World Health Organization (WHO) released a new classification that divided DF into two categories: severe and non-severe, to expedite the management of the disease [10,11]. There has been more than a 10-fold increase in the number of countries since 1970. KSA is one of them, and is now trying to control the increase in cases of DF every year [12].

In Jazan, 1970 cases were reported between 2005 and 2016, with a severe outbreak in 2016 (555 cases). There needs to be more health education about DF and the lifecycle of vectors among the Saudi population because of the few studies about the importance of health education on DF and the prevention measures to reduce the cases [13,14]. Therefore, the most crucial step to prevent the spread of DF infection, which is related to lifestyle, is avoiding causes, which requires KAP studies [1,13,14,15]. 

There is a large body of research that has investigated the level of knowledge that the general public has on dengue disease as well as the preventative methods and current situations. Research like this was carried out in Vietnam [5], Indonesia [16], Brazil [17], Yemen [18], Mexico, Colombia [19], Australia [20], and India [20]. Additionally, there are very few studies in the Middle East about the knowledge and compliance of the population with methods of prevention for dengue fever and the impact of campaigns on the population to combat DF, and there are few studies as far as we know regarding that in the Jazan region [2,13]. Therefore, the goal of this study was to use the KAP model and identify potential predictors to learn what the residents of the Jazan region know, feel, and do to prevent dengue fever, which is one of the most common diseases in this region.

## 2. Materials and Methods

### 2.1. Study Design and Participants

A cross-sectional study was conducted between April 2022 and May 2022 in the Jazan region, which lies in the southwest of Saudi Arabia on the Red Sea. The Jazan region covers an area of 13,457 km^2^, and the population is about 1.6 million, subdivided into 16 governorates. The study included all people who lived in the Jazan region. Both genders were considered targets for this study. Exclusion criteria were young people (<18 years old) and health workers.

### 2.2. Study Measures

A self-administered questionnaire designed by the WHO [4,21] was used in this study, with some modifications made to make it suitable for the study, to measure the KAP regarding DF among the population in the Jazan region. Knowledge refers to a community’s comprehension of dengue including its vectors and symptoms. Practices are the ways in which people demonstrate their attitude and knowledge through their behavior [21]. In this study, individual factors such as age, gender, education level, income, residence characteristics, and employment were independent variables, whereas the KAP scores were the dependent variable. The content validity of the scale was validated with the help of two different expert opinions. In order to determine the reliability of the scale, the Cronbach’s alpha was applied. The value was greater than 0.7, which is the threshold that is generally recognized as acceptable. The link to the questionnaire was distributed in public places for each participant across all governorates selected for this study. Then, an appropriate period was given to each participant to fill out the questionnaire. 

### 2.3. Sampling Technique and Sample Size

Convenient stratum random sampling was used in this study. The governorates were divided according to which areas were close to each other, and we chose a convenient stratum from them, and then we selected a random sample from each area as a study sample.

The sample size was calculated by using the following formula for random sampling:

*n* = *z* 2*pq*
*d* 2

where 

*n* is the calculated sample size;

*z* is the selected level of confidence = 1.96;

*p* is the estimated prevalence of knowledge in population = 50%;

*q* is (1 − *p*) = 50%; 

I is the maximum acceptable error [precision level] = 0.05; 

thus, *n* = (1.96^2^/0.5 × 0.5)/0.05^2^ = (3.8416 × 0.25)/0.0025 = 384.

### 2.4. Data Analysis

The collected data were interred into SPSS software version 23 (SPSS Inc., Chicago, IL, USA). The analysis included descriptive and inferential statistics as necessary. The means, standard deviation, and percentages were used properly. The investigation began with a review of the sample’s overall characteristics and descriptive statistics for the data variables. The frequency distributions for the categorical variables are then presented as well as the summary statistics for the continuous variables. The Pearson correlation coefficient was computed to assess the linear relationship between the three KAP constructs. The Shapiro–Wilk test was used to assure normality. After the sample was described, regression analysis was used to examine the adjusted correlations between the KAP and independent variables. Using multiple linear regression analysis, the impact of all independent variables on the KAP variables was investigated. Since the independent variables were categorical, they were incorporated into the models as dummy factors and relied on one of their classes as the reference group [22]. The beta coefficient (β) was used to estimate the correlations between the independent variables and the KAP variables, and a *p*-value of less than 0.05 was set as the threshold for significance. 

## 3. Results

### 3.1. Sample Characteristics

Table 1 shows a detailed description of the sample characteristics. A total of 392 participants were involved in this study. Among the 392 participants, 232 (59.18%) were male and 160 (40.82%) were female. Most participants (*n* = 299, 76.28%) were aged 18–35. Regarding education and employment status, about 72.96% of participants had a degree, and 38.01% were employed. Regarding the income distribution, 56.63% of the participants had a monthly income of less than SAR 5000.

### 3.2. Knowledge, Attitude, and Practice Scores among Participants

Table 2 shows the scores for knowledge, attitude, and practice concerning dengue fever among the study participants. The knowledge of the participants on dengue fever was represented by the knowledge score (similarly for attitude and practice). For the whole sample, the mean (±SD) knowledge, attitude, and practice score of the respondents were 22.77 ± 7.9, 22.68 ± 7.24, and 25.62 ± 9.4, respectively. Table 2 also displays the knowledge, attitude, and practice scores for dengue fever among the study participants. The knowledge and attitude scores were higher among males (23.73 ± 8.22, 23.97 ± 6.61, respectively), while female participants showed higher practice scores (26.88 ± 8.65). Participants who lived in cities showed high knowledge, attitude, and practice scores (22.76 ± 8.03, 23.05 ± 7.3, and 26.25 ± 9.05, respectively). Participants with higher education attainment have higher knowledge and attitude scores than those with no degree (23.65 ± 7.89, 23.2 ± 7.08, respectively), while participants with no degree showed higher practice scores (26.27 ± 9.08). Employed participants showed higher knowledge, attitude, and practice scores than the unemployed participants (23.49 ± 7.25, 23.56 ± 6.27, and 26.24 ± 9.88, respectively). Regarding income, participants with high income ≥SAR 15,000 showed high knowledge and practice scores (25.4 ± 7.62 and 26.4 ± 10.2, respectively), and those with income between SAR 5000–9999 (USD 1 = 3.76) showed high attitude scores (24.03 ± 7.2). Participants aged between 56 and 70 years showed high attitude and practice scores (22.86 ± 9.06 and 29.29 ± 6.73, respectively), while participants aged between 18 and 35 showed high knowledge scores (22.83 ± 8.35).

### 3.3. Pearson’s Correlation Analysis of Knowledge, Attitude, and Practice Scores

**Figure 1:**
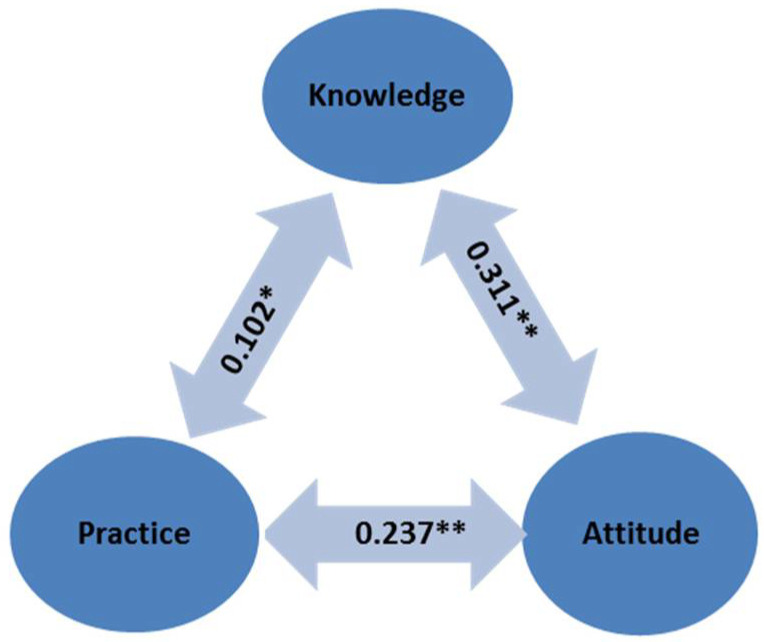
Pearson’s correlation analysis of the KAP constructs. Values in the double head arrows are the Pearson’s correlation coefficients (r). ** Correlation was significant at the 0.01 level (2-tailed). * Correlation was significant at the 0.05 level (2-tailed).

A Pearson correlation coefficient was computed to assess the linear relationship between the three KAP constructs (Figure 1). There were positive correlations between the three variables (*p* < 0.05). The attitude and knowledge scores showed the strongest correlation between these variables r(392) = 0.311, *p* < 0.01.

### 3.4. The Association between KAP Scores and Sociodemographic Characteristics

Multiple linear regression analysis was performed to examine the relationship between knowledge, attitude, and practice variables and sociodemographic characteristics in the sample. Being male was positively and significantly associated with the attitude score (β = 2.76, *p* = 0.001), and negatively associated with the practice score (β = −2.45, *p* = 0.023). Participants with no degree (bachelor/master or PhD) had a lower knowledge score compared to individuals with a degree (β = −2.78, *p* = 0.003). Finally, having an income more than SAR 15,000 and between SAR 5000–9999 were associated with higher knowledge score (β = 4.11, *p* = 0.006, β = 3.01, *p* = 0.011, respectively). The rest of the associations are summarized in Table 3.

## 4. Discussion

DF is a viral infection transmitted through infected mosquito bites that can lead to morbidity, hospitalization, and, in some cases, death [3,23]. In addition, 15,369 patients (59.7%) out of 25,745 declared cases in the KSA were estimated to have suffered from DF between 2013 and 2017, resulting in an average cost of USD 11,947.6 per patient [24]. This study aimed to measure the KAP toward DF and its control measures among the Jazan population in the southwest of Saudi Arabia. 

According to this study, participants aged 15 to 35 who had higher education, were working, or earned salaries of SAR 5000 to 9999 (USD 1 = 3.76) or more per month or more than SAR 15,000 had greater knowledge than those who were younger, did not have a degree or job, or earned lower salaries. This concurred with a different cross-sectional study conducted in Indonesia [25]. Albasheer O.B. et al. (2020) found a different relationship between the participants’ age and knowledge, with the participants’ knowledge levels being significantly influenced by their age group of more than forty years [2]. The attitude scores of 56–70-year-old males with higher education, employment, or a salary between SAR 5000 and 9999 were higher than those of younger-aged females without a degree, job, or lower salaries. Participants aged 56–70, without a degree, employed or earning over SAR 15,000 had higher practice scores than those younger and with no degree, job, or lower salaries.

Most of the studies in the KSA regarding the KAP of DF were conducted among secondary or high school students [14,26,27], except for one study that was carried out on primary health care participants in the Jazan region [2]. However, it is hard to compare our results to those of the study by Albasheer, O.B. et al. because the participants in that study were people who went to primary health care clinics, and they used a different KAP scoring system. Some similar questions from the study by Albasheer O.B. et al. (2020) and other international studies can be summarized as follows: 70% of the participants were from the 25- to 34-year-old group, which was similar to our study. Albasheer, O.B. et al. (2020) and other international studies found that about 70% of participants correctly identified the causative agent (mosquito bites and not transmitted through direct person-to-person contact) when asked about their knowledge [2,26,27,28]. Furthermore, more than 70% of the participants correctly answered the question related to the symptoms of dengue fever [2,28]. Regarding attitude, around 80% of participants agreed that DF is a serious disease and that prevention and control are the government’s responsibility. 

Regarding preventive measures, it is essential to note that the vaccine does not reduce dengue fever in areas where it is prevalent. In order to prevent dengue fever from spreading, mosquito bite prevention and mosquito control must be implemented [4]. In the same study by Albasheer, O.B. et al. (2020), more than half of the participants agreed to put nets on the windowpanes, dispose of water collection containers, and cover exposed body parts [2]. 

The KAP factors and sociodemographic aspects were examined using multiple linear regression. The findings revealed that non-degree participants obtained poorer knowledge scores than degree holders. A considerable favorable correlation between education and excellent knowledge on DF was found to exist, as supported by a number of studies [29,30,31]. This might be due to the fact that persons with higher levels of education are more likely to participate in health awareness campaigns and educational initiatives as well as have access to more information regarding DF. There was an obvious and clear connection between economic position (a higher income) and having a strong understanding of DF [30,31,32,33]. This association exists in the current research. People with higher economic status may have better access to credible knowledge as well as a greater sense of its value. Other research has found substantial connections between having a high knowledge score of dengue and being employed, growing older, and being female [30,33]. 

The mean scores of knowledge, attitude, and practice were fairly correlated in these self-reported questions. The participants’ attitudes and behavior in the research area improved as their knowledge grew [25]. Additionally, a substantial positive correlation between knowledge, attitude, and practice was found. Our findings were most comparable to some past research. Only a link between the knowledge of dengue and a favorable attitude toward dengue control and between a positive attitude and effective dengue preventative practices was discovered in other investigations [31,34]. However, this study still needs to translate information more effectively into community attitudes and practices. Even though the study population had a wealth of knowledge, they were not always moral in their actions or thoughts [25,35]. There is conflicting evidence that knowledge and practice are related. While some people noticed a connection between good knowledge and behaviors in Cuba [36], Malaysia [21], and Laos [37], others did not. Our present research offers a method for transforming community knowledge into lasting, beneficial habits. 

Our study has several strengths. First, we investigated the KAP of DF in the most prevalent city population. Second, we used the most updated WHO questions to inform the questionnaire. Finally, we used a random sample to decrease the selection bias. The limitations of this study include that it was conducted in one city and that most of the participants had degrees but were unemployed. This may not represent all Saudis, making generalization of the study’s results difficult.

## 5. Conclusions

The study revealed multiple hideouts regarding the knowledge, behavior, and practices that benefit or harm citizens. Demographic factors showed a different effect on these three factors. Policymakers have the opportunity to design measures to raise awareness, understanding, and surveillance through the use of community-based educational initiatives. It is possible that more study could be conducted in Saudi Arabia to make the findings more widespread and to mitigate the consequences of dengue outbreaks.

## Figures and Tables

**Table 1:** Sample characteristics (*n* = 392).

Characteristics	Frequency (%)
**Gender**	
Male	232 (59.18%)
Female	160 (40.82%)
**Age (years)**	
“18–35”	299 (76.28%)
“36–55”	86 (21.94%)
“56–70”	7 (1.79%)
**Education**	
Has Degree	286 (72.96%)
No Degree	106 (27.04%)
**Residence**	
City	172 (43.88%)
Village	220 (56.12%)
**Income (Saudi Riyal)**	
“≤4999”	222 (56.63%)
“5000–9999”	72 (18.37%)
“10,000–14,999”	48 (12.24%)
“≥15,000”	50 (12.76%)
**Work**	
Employed	149 (38.01%)
Unemployed	243 (61.99%)

Abbreviations: SD: standard deviation, *n*: sample size.

**Table 2:** Knowledge, attitude, and practice scores of the participants in the sample.

Variables		Knowledge	Attitude	Practice
		(Mean ± SD)		
Gender	Male	23.73 ± 8.22	23.97 ± 6.61	24.76 ± 9.81
	Female	21.38 ± 7.2	20.81 ± 7.71	26.88 ± 8.65
Age	“18–35”	22.83 ± 8.35	22.76 ± 7.78	25.28 ± 9.21
	“36–55”	22.62 ± 6.45	22.38 ± 4.77	26.51 ± 10.15
	“56–70”	22.14 ± 2.67	22.86 ± 9.06	29.29 ± 6.73
Education	Has Degree	23.65 ± 7.89	23.2 ± 7.08	25.38 ± 9.52
	No Degree	20.38 ± 7.42	21.27 ± 7.5	26.27 ± 9.08
Residence	City	22.76 ± 8.03	23.05 ± 7.3	26.25 ± 9.05
	Village	22.77 ± 7.81	22.39 ± 7.19	25.14 ± 9.65
Income *	“≤4999”	21.58 ± 7.86	21.98 ± 7.87	25.54 ± 9.08
	“5000–9999”	24.24 ± 8.42	24.03 ± 7.2	24.86 ± 10.1
	“10,000–14,999”	23.33 ± 6.55	22.71 ± 5.83	26.35 ± 9.04
	“≥15,000”	25.4 ± 7.62	23.8 ± 5.01	26.4 ± 10.2
Work	Employed	23.49 ± 7.25	23.56 ± 6.27	26.24 ± 9.88
	Unemployed	22.33 ± 8.25	22.14 ± 7.74	25.25 ± 9.09
Whole Sample N = 392		22.77 ± 7.9	22.68 ± 7.24	25.62 ± 9.4

* USD 1 = SAR 3.76.

**Table 3:** Linear regression analysis of the association between the KAP scores and sociodemographic characteristics.

Variables	Levels	Knowledge (β + *p* Value)	Attitude (β + *p* Value)	Practice (β + *p* Value)
Gender (Reference: Female)	Male	1.29 (0.144)	2.76 (0.001) *	−2.45 (0.023) *
Age (Reference: “18–35”)	“36–55”	−0.66 (0.557)	−0.53 (0.610)	0.28 (0.839)
	“56–70”	1.44 (0.637)	2.03 (0.472)	1.88 (0.615)
Education (Reference: Has Degree)	No Degree	−2.78 (0.003) *	−1.34 (0.123)	0.44 (0.698)
Residence (Reference: City)	Village	0.22 (0.781)	−0.74 (0.310)	−0.86 (0.373)
Income (Reference: “≤4999”)	“5000–9999”	3.01 (0.011) *	1.15 (0.292)	−0.82 (0.567)
	“10,000–14,999”	2.15 (0.157)	−0.33 (0.812)	0.09 (0.963)
	“≥15,000”	4.11 (0.006) *	0.54 (0.690)	0.24 (0.894)
Work (Reference: Employed)	Unemployed	1.23 (0.280)	−0.32 (0.764)	−1.81 (0.192)

Abbreviations: KAP: knowledge, attitude, and practice, B: beta coefficient, * *p* value < 0.05.

## Data Availability

Data are available upon request due to ethical restrictions regarding participant privacy. Requests for the data may be sent to the corresponding author.
